# Carnitine Palmitoyltransferase II (CPT2) Deficiency: An Overlooked and Elusive Cause of Acute Kidney Injury

**DOI:** 10.7759/cureus.70442

**Published:** 2024-09-29

**Authors:** Saimir Seferi, Kristi Saliaj, Eriola Likaj, Merita Rroji

**Affiliations:** 1 Department of Nephrology, University Hospital Center "Mother Teresa", Tirana, ALB; 2 Department of Nephrology, University Hospital Center “Mother Teresa”, Tirana, ALB

**Keywords:** acute kidney injury (aki), cpt2 deficiency, dialysis, myopathy, rhabdomyolysis

## Abstract

Carnitine palmitoyltransferase II (CPT2) deficiency is a rare inherited disorder affecting fatty acid metabolism. This enzymatic defect presents with a broad clinical spectrum, from severe neonatal forms that can be fatal, to milder myopathic variants characterized by myalgia and recurrent myoglobinuria in adolescence and adulthood. Herein, we report the case of a male patient who developed exertional rhabdomyolysis and acute kidney injury due to CPT2 deficiency.

This case underscores the importance of considering genetic disorders in the differential diagnosis of patients presenting with recurrent exercise intolerance and metabolic crises. Early recognition and diagnosis enable prompt implementation of dietary and lifestyle modifications aimed at mitigating potential complications such as renal impairment. Moreover, timely diagnosis allows for genetic counseling of affected individuals and their families.

## Introduction

Fatty acids are an important energy source, especially during periods of increased energy demand and low glucose availability, such as fasting or febrile illness. During these times, the body increases the mobilization of triglycerides into free fatty acids and glycerol, which subsequently undergo beta-oxidation in the mitochondria to produce adenosine triphosphate (ATP) [1,2]. However, long-chain fatty acids cannot readily pass through the mitochondrial membrane. They are assisted by the carnitine palmitoyltransferase (CPT) system, an integral enzymatic shuttle that facilitates their transport into the mitochondrial matrix [1-4].

The CPT system includes two key enzymes: CPT1 and CPT2, located in the outer and inner mitochondrial membranes, respectively [3-5]. CPT1 has three isoforms, expressed in muscle, liver, and brain, whereas CPT2 is ubiquitously [3-5]. Mutations in the genes encoding CPT1 and CPT2 can lead to rare and often severe metabolic disorders [3-5].

CPT2 deficiency is a rare disorder characterized by an impaired ability to oxidize long-chain fatty acids [3-5]. It is inherited in an autosomal recessive pattern, with an estimated prevalence of 1-9/100,000 people [5,6]. It primarily shows a male tendency [5]. Higher levels of estrogen that modulate hepatic lipid metabolism, modifier genes on chromosome X, or gender differences in exercise habits may contribute to this predilection [5,7]. Differential diagnosis of metabolic myopathies is broad and includes defects in fatty acid oxidation, glycogen storage disease, and mitochondrial myopathies that disrupt normal energy production. Important conditions to rule out include McArdle disease, Duchenne muscular dystrophy, and very-long-chain acyl-coenzyme A dehydrogenase deficiency.

Clinical presentation is heterogeneous, with manifestations ranging from hypoketotic hypoglycemia, muscle weakness, and myoglobinuria, to systemic involvement with multiple organ failure [3-5].

## Case presentation

A 33-year-old male, previously asymptomatic, presents to the Emergency Department with a three-day history of fatigue, nausea, muscle weakness, myalgia, and dark-colored urine, as well as a progressive decline in urine output after strenuous exercise. His past medical history was unremarkable. His social history was significant for moderate alcohol consumption. However, he reported that his older brother, experienced similar symptoms six years ago, following a short period of intense physical activity. His brother had previously experienced recurrent episodes of myalgias and dark-colored urine during childhood, as well.

On physical examination, he appeared unwell. Lower limb muscle tenderness and mild peripheral edema were present, with no signs of erythema or other musculoskeletal anomalies. His neurological examination was within normal limits and the rest of his physical examination was unremarkable. On presentation, he was anuric.

**Figure 1:**
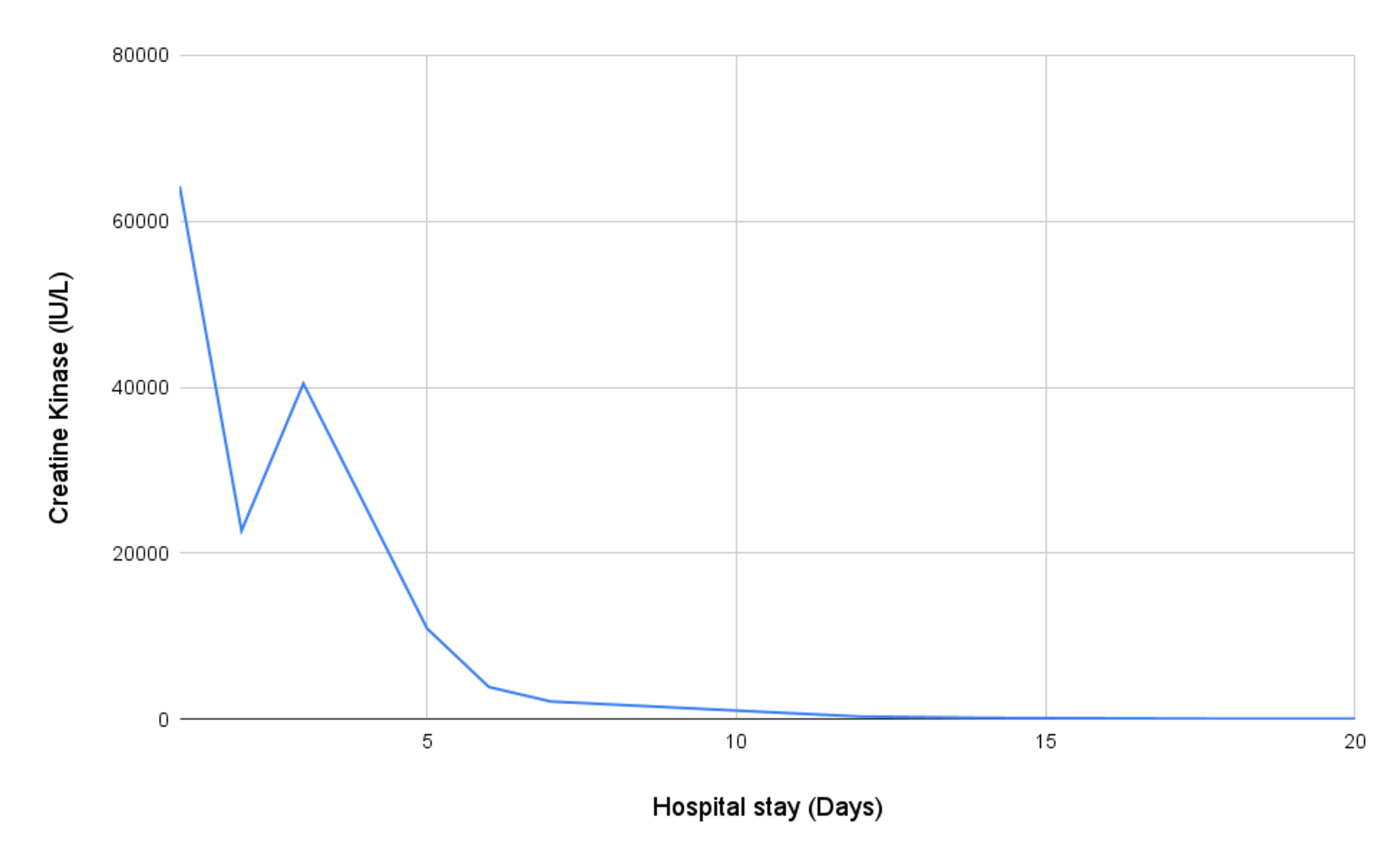
Creatine kinase levels during the hospital stay

A comprehensive metabolic panel revealed elevated serum creatine kinase (CK) 64,180 IU/L (normal range 55 - 170 IU/L), creatine kinase - MB (CK-MB) 1057 IU/L (normal range 5- 25 IU/L) and lactate dehydrogenase (LDH) 2890 IU/L (normal range 140- 280 IU/L) levels, abnormal liver function tests with elevated aspartate aminotransferase (AST) 4017 IU/L (normal range 8- 33 IU/L), alanine aminotransferase (ALT) 1000 IU/L (normal range 10- 50 IU/L) levels, and acute kidney injury (AKI) with serum creatinine 6.6 mg/dL, urea 169 mg/dL. A diagnosis of exertional rhabdomyolysis associated with AKI was established (Figure 1).

**Figure 2:**
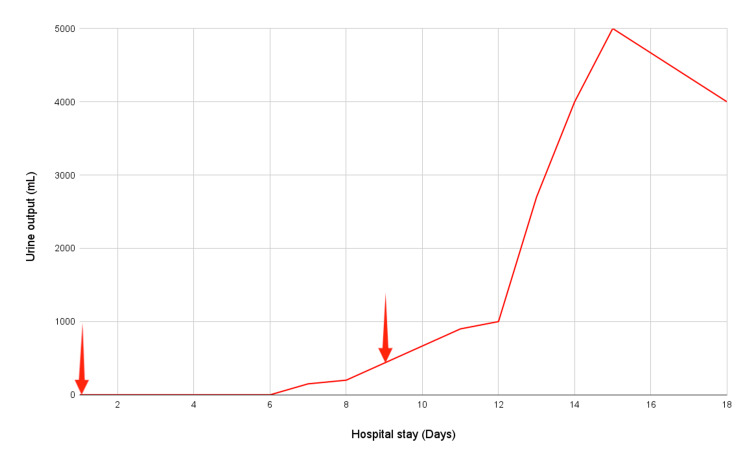
Urine output during the hospital stay

The patient was started on IV fluid therapy, however on account of his persistent anuria despite fluid resuscitation following an initial 12-hour period; a decision to initiate dialysis was made. He underwent eight hemodialysis sessions in total. His kidney function recovered completely and he was weaned off dialysis (Figure 2).

In light of his suggestive family history, a genetic work-up was ordered to evaluate for a potential hereditary condition. Next- generation sequencing (NGS) revealed a pathogenic variant involving the CPT2 gene on chromosome 1p32, a missense mutation leading to a single amino acid change from serine to leucine. NGS showed his brother carried the same homozygous mutation, as well. 

At discharge, the patient made a full recovery. He was advised to incorporate in his diet carbohydrate-rich food and avoid long-chain fatty sources and potential triggers.

## Discussion

Fatty acid β-oxidation (FAO) represents a vital bioenergetic pathway, particularly during periods of high-energy demand and metabolic stress when glucose is scarce [3,5,8]. Long-chain fatty acids (LCFA) serve as substrates in a cascade leading to the synthesis of acetyl-coenzyme A (acetyl-CoA), which then enters the tricarboxylic acid cycle (TCA cycle) to generate ATP [3,5,8-10]. β- oxidation occurs primarily in the mitochondrial matrix and is mediated by the CPT system, a crucial enzymatic system for translocating LCFA across the mitochondrial membrane [3,5,8-10].

This pathway is essential during strenuous conditions and limited glucose availability, such as infections, intense exercise, fasting, or cold exposure [3,5,8-10]. Defects in the CPT system impair normal lipid and glucose metabolism and disrupt energy homeostasis [3,5,8-10].

Clinical presentation encompasses three distinct phenotypes: fatal neonatal-onset characterized by cerebral malformations, seizures, hypotonia, cardiomyopathy, and circulatory failure; infantile form with features of multisystem involvement and early death; adult-onset: the most common form, characterized by muscle pain and myoglobinuria [8-13].

It is the most common inherited disorder of LCFA oxidation and the leading cause of recurrent myoglobinuria in adults [4,5]. The adult-onset myopathic phenotype is characterized by muscle pain, weakness, and myoglobinuria [8-13]. Recurrent episodes of rhabdomyolysis and myoglobinuria can lead to AKI and respiratory failure, often triggered by intense or prolonged exercise, fasting, infection, and cold exposure [4,5,8-13]. CPT2 activity is less than 10% in the neonatal and infantile phenotype and less than 30% in the adult-onset form [12]. Most patients don’t report cramps with prolonged physical activity, which signals impending muscle injury [14]. By the time muscle pain and stiffness develop myoglobinuria and often kidney injury has already been established.

Between attacks patients with CPT2 deficiency generally do not experience symptoms of myopathy, myalgia or muscle weakness, and show no abnormal laboratory findings, including normal serum CK and carnitine levels [4,5,8-13]. Approximately 10% of patients exhibit persistent myopathy [5]. Diagnostic workup may include measuring enzymatic activity in skeletal muscle biopsy, assessing serum acylcarnitine profiles showing increased C12 to C18 acylcarnitine levels and specifically (C16 + C18:1)/C2 ratio, highly suggestive of CPT2 deficiency [13]. Nevertheless, definitive diagnosis requires genetic testing, either single gene or multipanel testing, to exclude other hereditary diseases [13].

The condition arises due to homozygous and compound heterozygous mutations affecting the CPT2 gene, located on the short arm of chromosome 1 [14-16]. The Ser113Leu mutation accounts for the majority of cases, present in more than 50% of patients with the myopathic phenotype [14-16]. However, other missense mutations, including P50H, F448L, R503C, and G549D, have also been found to be pathogenic, highlighting the molecular heterogeneity of the disease [14,15].

Studies show that missense mutations are more commonly associated with the milder, adult-onset myopathic variant, in contrast to truncating and frameshift mutations, which are associated with the multisystemic neonatal and infantile phenotypes [14]. The disease follows an autosomal recessive inheritance pattern; however, compound heterozygosity for a missense and truncating mutation can result in either mild or severe phenotypes [14]. Research suggests that heterozygosity for a single mutation may be associated with reduced enzymatic activity, placing carriers at a higher risk of developing clinical symptoms or symptomatic disease [14,15].

Our patient presented with a family history suggestive of hereditary rhabdomyolysis, prompting further investigations. Clinical presentation aligned with CPT2 deficiency, with symptoms of muscle pain, weakness, and lower limb tenderness, dark-colored urine, a progressive decline in urine output, and AKI, triggered by intense physical activity. Genetic testing confirmed the presence of a pathogenic homozygous mutatation in the CPT2 gene that was subsequently found to be carried by his brother, as well. As the enzymatic deficiency is inherited in an autosomal recessive pattern, each sibling carries a 25% chance of inheriting the mutation and being affected by the condition. The fact that both siblings were affected and presented with clinically relevant manifestations of the disease highlights the rarity of this case report. Additionally it underscores the importance of considering genetic mutations in the differential diagnosis of rhabdomyolisis.

Treatment strategies vary based on clinical severity. During episodes of transient myalgia and myoglobinuria, supportive care with fluid therapy and bedrest are paramount and lead to a full recovery [4,5,8-13]. On the other side of the spectrum, severe presentations with rhabdomyolysis, AKI or respiratory failure warrant aggressive management including IV fluid resuscitation and initiation of renal replacement therapies [4,5,8-13]. Glucose administration is preferred to provide the adequate carbohydrate substrate to prevent adipose tissue lipolysis and improve the metabolic crisis [13]. According to the official recommendations of the British Inherited Metabolic Disease Group, treatment involves correcting dehydration with normal saline (0.9%) and addressing hypoglycemia, if present, with 50 mL of 50% dextrose over 30 minutes [17]. Following this, intravenous 10% dextrose should be administered at a rate of 2 mL/kg/hr [17]. If hyperglycemia develops, an insulin infusion should be initiated according to local protocols [18]. Fluid therapy should be adjusted based on the patient's volume status, with the goal of maintaining a urine output of 200-300 mL/hr, in accordance with rhabdomyolysis guidelines, while avoiding volume overload [13]. Intravenous fluids should be continued until creatine kinase (CK) levels drop below 5000 IU/L and continue to decline.

Initiation of renal replacement therapies is often required in the context of oliguria, refractory metabolic acidosis and hyperkalemia, as well as diuretic-refractory volume overload [13].

Currently, no specific treatment exists for CPT2 deficiency. Carnitine supplementation has not demonstrated improved outcomes and has recently been discouraged due to potential cardiovascular risks [18]. Supplementation with medium chain triglycerides (MCT), creatine, essential aminoacids (EAAs), vitamin D to support bioenergetics and lipid metabolism in muscle tissue, as well as omega-3 polyunsaturated fatty acids (PUFA) and antioxidant-rich foods to promote muscle recovery, are proposed interventions, but further research is needed to confirm their efficacy [18]. Some case reports and small trials suggest that treatment with bezafibrate, a hypolipidemic medication, has been associated with improved beta-oxidation, enzyme activity, and myalgia, as well as a reduction in rhabdomyolysis episodes and an increase in physical activity [5,19]. However, rhabdomyolysis episodes may still occur in response to known triggers, and these findings need to be corroborated in larger populations [19].

Avoidance of potential triggers, metabolic stressors and adherence to a strict low-fat (<20%) and carbohydrate rich (>70%) diet, remain the cornerstone of CPT2 deficiency management.

## Conclusions

CPT2 deficiency is a rare metabolic disorder manifesting across a spectrum of phenotypes. The adult-onset myopathic variant is an important cause of recurrent myoglobinuria. Diagnosis can be challenging as the clinical presentation is often ambiguous, thus a high index of suspicion is warranted. Despite the lack of targeted therapies, the implementation of dietary modifications and avoidance of precipitating factors are straightforward, yet effective management strategies leading to long-term favorable outcomes.
